# Understanding change in COVID-19 vaccination intention with network analysis of longitudinal data from Dutch adults

**DOI:** 10.1038/s41541-022-00533-6

**Published:** 2022-10-01

**Authors:** Monique Chambon, Wesley G. Kammeraad, Frenk van Harreveld, Jonas Dalege, Janneke E. Elberse, Han L. J. van der Maas

**Affiliations:** 1 National Institute for Public Health and the Environment (RIVM), Antonie van Leeuwenhoeklaan 9, 3721 MA Bilthoven, The Netherlands; 2 Department of Psychology, University of Amsterdam, Nieuwe Achtergracht 129-B, 1018 WS Amsterdam, The Netherlands; 3 Santa Fe Institute, 1399 Hyde Park Road, Santa Fe, NM 87501 USA

**Keywords:** Infectious diseases, Epidemiology

## Abstract

Prior research into the relationship between attitudes and vaccination intention is predominantly cross-sectional and therefore does not provide insight into directions of relations. During the COVID-19 vaccines development and enrollment phase, we studied the temporal dynamics of COVID-19 vaccination intention in relation to attitudes toward COVID-19 vaccines and the pandemic, vaccination in general, social norms and trust. The data are derived from a longitudinal survey study with Dutch participants from a research panel (*N* = 744; six measurements between December 2020 and May 2021; age 18–84 years [*M* = 53.32]) and analyzed with vector-autoregression network analyses. While cross-sectional results indicated that vaccination intention was relatively strongly related to attitudes toward the vaccines, results from temporal analyses showed that vaccination intention mainly predicted other vaccination-related variables and to a lesser extent was predicted by variables. We found a weak predictive effect from social norm to vaccination intention that was not robust. This study underlines the challenge of stimulating uptake of new vaccines developed during pandemics, and the importance of examining directions of effects in research into vaccination intention.

## Introduction

Managing the worldwide COVID-19 pandemic through preventive behaviors aimed at preventing the spread of the virus entered a new phase when vaccines became available. An important aspect of disease protection through vaccines is that collective (group) protection requires a minimum proportion of people obtaining the vaccination. Vaccination uptake however is far from self-evident, as vaccines are often the subject of controversy. Vaccine hesitancy is defined as a “delay in acceptance or refusal of vaccination despite availability of vaccination services”^1^. Although most people report intention to get vaccinated against COVID-19, vaccine hesitancy is also widespread: in the first half of 2020 only around three-quarters of respondents from Europe and the United States reported willingness to get vaccinated against COVID-19^2,3^. Moreover, longitudinal research conducted before the vaccines were rolled out observed a decrease in vaccination intention^4,5^. Since the rollout, most studies report an increase in vaccination intention over time^6–8^. Improving our understanding of people’s intention to get vaccinated can help to stimulate vaccine uptake. Such insights are relevant not only for the COVID-19 pandemic but also for potential future pandemics with newly developed vaccines.

Prior research states that the largest obstacles to the successful implementation of COVID-19 vaccines are attitudinal—that is, vaccination acceptance is impeded by concerns about potential side effects, and a lack of trust in the safety and benefits of the vaccine^2,9–13^. Prior work on attitudes demonstrates challenges that generally accompany attitude change and indicates that understanding attitude structure is beneficial to the design of effective attitude change strategies^14,15^. However, vaccine uptake is not exclusively explained by attitudes toward the vaccine. Psychological variables such as trust in science and social norms in favor of getting vaccinated also showed a positive relation with (intended) vaccine uptake^16,17^. This suggests that understanding vaccination intention requires a broad perspective that includes not only attitudes but also other psychological variables. Psychological variables that are expected to be relevant for attitudes toward COVID-19 vaccines and vaccination intention are attitudes toward the COVID-19 pandemic^18^, and vaccines in general^19^. Therefore, in our study, we included beliefs^10,20^, affective responses^21^, trust^16,22^, social norms^16,23^, and self-reported adherence to protective guidelines regarding social distancing and hygiene.

Although previous research demonstrates that attitudes and related variables are important for the intention to get vaccinated against COVID-19, little is known about the trajectory in which these attitudes and vaccination intentions develop. Review studies on factors associated with vaccination showed that prior research is predominantly cross-sectional^24–27^, and thus cannot provide insight into the direction of effects between variables relevant for vaccination. The majority of previous longitudinal research into the relation between attitudes and COVID-19 vaccination seems to rely on two measurements^28,29^, therefore not providing sufficient detail for analysis techniques into predictive effects such as employed in the current study.

Furthermore, given that COVID-19 vaccines were yet to be developed, attitudes toward the vaccines emerged during the pandemic as well. This provided a unique opportunity to study the temporal dynamics of attitudes toward newly developed vaccines. Accordingly, this study was conducted during the phase of the COVID-19 pandemic in which vaccines were developed and rolled out in the Netherlands (December 2020 to May 2021; six measurements). Studying attitudes during this phase may enable us to capture attitude development before attitudes polarize, as is often the case with topics such as vaccination.

Given this study’s broad approach, including a range of variables that are expected to interconnect, we adopted a complex systems perspective on attitudes to shed light on how these variables interact over time. This approach is based on the Causal Attitude Network (CAN) model^30^ that defines attitudes as psychological attitudinal networks consisting of attitudinal elements—affect, cognition, and behavior—with causal interactions explicable through a network topology. Attitude networks consist of evaluative reactions (nodes) and interactions between them (edges, i.e., linear relations between nodes). Edges represent either excitatory or inhibitory influence with varying weights (i.e., the causal influence between evaluative reactions varies). The CAN model conceptualizes the system’s overall state (i.e., pattern of thoughts, feelings, and behaviors of a person) as arising from interactions between attitude elements^30^.

**Fig. 1:**
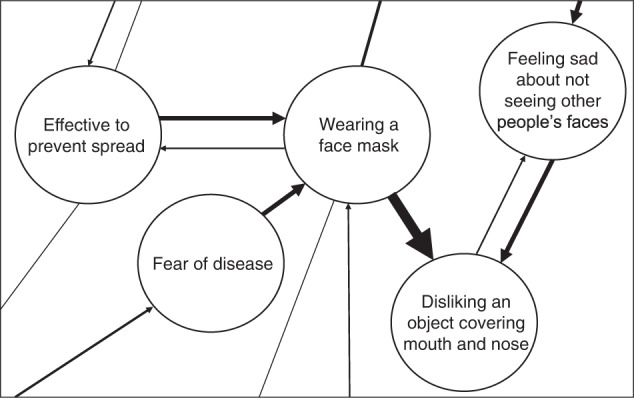
Hypothetical, simplified network of the attitude toward wearing a face mask. The network consists of a behavioral element (“Wearing a face mask”), cognitive element (“Wearing a face mask is effective to prevent the spread of the virus”), and three affective elements (“Fear of disease caused by the virus”, “Feeling sad about not seeing other people’s faces” and “Disliking an object covering mouth and nodes”). Edges with arrows indicate the direction of predictive effects and the width indicates the strength of this effect. This example shows that disliking an object covering mouth and nose is predicted by both wearing a face mask and feeling sad about not seeing other people's faces, with the former having the strongest predictive effect, as indicated by different edge width. The example shows both unidirectional (e.g., wearing a face mask and fear of disease) and bidirectional effects (e.g., wearing a face mask and effective to prevent spread) with edges of different strengths.

Attitude network models can be fitted to data^31^. Patterns of interactions can be identified as partial correlations in which an interaction between two nodes is conditional on every other node in the network, e.g., see^32,33^. In other words, psychometric networks provide an integrated summary of unique statistical relations between variables (i.e., relations that cannot be explained by other variables in the network), which is presented in a clear and visually attractive manner and can be theoretically interpreted in the CAN model. Figure 1 depicts a hypothetical, simplified network of the attitude toward wearing face masks during a pandemic.

The current study extends the CAN model by including a broader range of variables relevant to attitudes toward COVID-19 vaccines and vaccination intention, resulting in a broad attitude network approach. Moreover, its longitudinal design enables examining predictive effects of variables in the broad COVID-19 vaccines network during the unique phase of development and enrollment of the vaccines. Such temporal data reveal important clues about causal relations between variables in the network and allow distinguishing between within- and between-person effects.

## Results

**Fig. 2:**
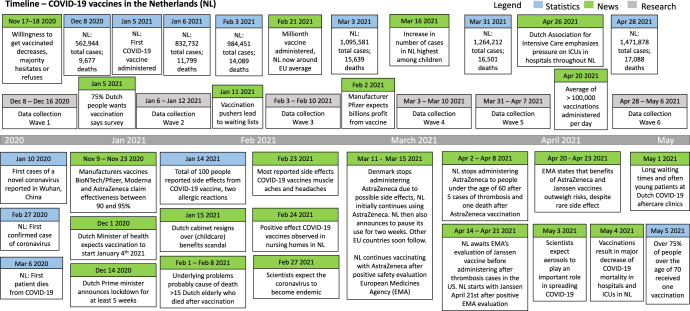
Dutch COVID-19 vaccines timeline. Timeline of Dutch media coverage on COVID-19 vaccines and the pandemic’s trajectory in the Netherlands.

The current section presents the temporal dynamics of attitudes and behaviors toward COVID-19 vaccines. Results are best interpreted in their context; therefore, Fig. 2 provides a global overview of Dutch media coverage on COVID-19 vaccines and the pandemic’s trajectory in the Netherlands (see Supplementary Note 0 for data). The political system of the Netherlands can be characterized as a typical western democracy.

### Descriptive results

The longitudinal sample consisted of 52% females and age at the last measurement ranged from 18 to 84 years (*M* = 53.32, SD = 15.25). In total, 60.1% reported primary or secondary education as the highest completed degree, whereas 39.9% reported completing higher education. The mean score on general health was 3.70 (SD = 0.79), indicated on a 5-point Likert scale from 1 (Very bad) to 5 (Very good). A total of 8.7% reported to have been infected with COVID-19 (with or without test confirmation), 77.8% reported no infection, and 13.4% does not know.

**Fig. 3:**
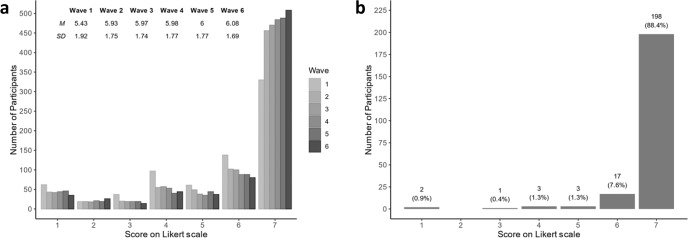
Descriptive results of COVID-19 vaccination intention. **a** Distribution of respondents’ scores on COVID-19 vaccination intention for each wave. Table with mean (M) and standard deviation (SD) per wave for COVID-19 vaccination intention score inserted above. **b** Last known score on COVID-19 vaccination intention (displayed on X axis) of participants who reported to have been vaccinated against COVID-19 (*n* = 224).

Figure 3a shows the number of respondents per score on COVID-19 vaccination intention for each wave. Node-specific descriptive statistics for each measurement (i.e., survey in a wave) are provided in Supplementary Note 1. Table 1 depicts changes in COVID-19 vaccination intention on an individual level for each wave, indicating the stability of respondents’ answers. During the first wave, the change in vaccination intention was predominantly positive (i.e., an increase in vaccination intention), whereas the change in vaccination intention during later waves was both positive and negative. Supplementary Note 2 provides an overview of changes in vaccination intention divided based on the demographic information of gender and age. These results suggest that women and younger participants (i.e., aged below the median age of the sample) changed their score on vaccination intention more often.

**Table 1:** Number of respondents that changed their score on the 7-point Likert answer scale for COVID-19 vaccination intention for each wave.

		Change in score for COVID-19 vaccination intention												
		−6	−5	−4	−3	−2	−1	0	1	2	3	4	5	6
Waves	T1 –> T2	0	0	0	3	9	29	435	165	66	24	11	1	1
	T2 –> T3	0	1	1	7	13	65	547	78	22	9	0	1	0
	T3 –> T4	0	1	2	6	11	64	570	70	14	4	0	2	0
	T4 –> T5	0	1	1	4	12	63	571	64	22	5	1	0	0
	T5 –> T6	1	0	0	2	12	37	599	65	21	4	1	1	1

From wave 5 onwards, vaccination became available and respondents indicated whether they had been vaccinated. In total, 224 respondents indicated they received a COVID-19 vaccine. Figure 3b displays their scores on vaccination intention in the wave prior to the wave in which they indicated to have been vaccinated against COVID-19. Note that during these measurements a large proportion of the Dutch public (i.e., age under 60 years without medical condition) had not yet received an invitation to get vaccinated against COVID-19, therefore it is not possible to reliably calculate the relation between vaccination intention and actual behavior.

### Network results

#### Preliminary analyses

The pruned network model, including only edges that are significant at *a* = 0.05, showed the best fit with the observed data and is thus presented here. Overall fit of the pruned *panelgvar* model was excellent according to RMSEA (root mean square error of approximation = 0.04 [95% CI 0.04–0.05]) and CFI (comparative fit index = 0.96). Additional fit measures are provided in Supplementary Note 3. Confidence intervals of edge weights were generally not wide, indicating reliable (stable) edge estimates. A complete overview of edges in the broad COVID-19 vaccines networks is provided in Supplementary Note 4 (i.e., edge weight tables and figures with edge weight confidence intervals).

#### Between-person broad COVID-19 vaccines network

**Fig. 4:**
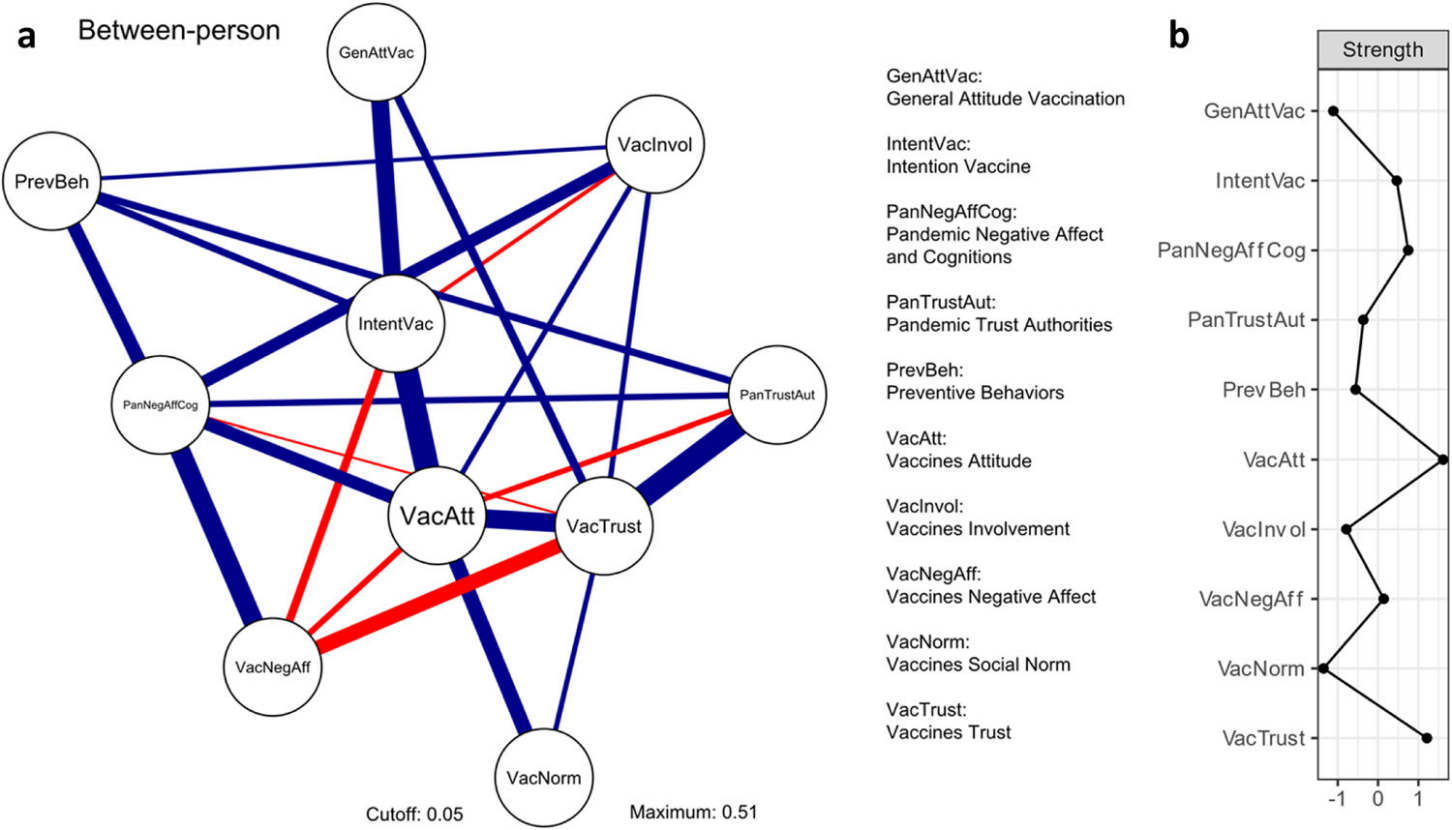
Between-person broad COVID-19 vaccines network. **a** Network: nodes represent measured variables. Edges indicate partial correlations after controlling for every other node in the network. Edge weights, as indicated by width, represent the strength of relations (i.e., correlation coefficient). Blue edges represent positive (excitatory) effects that indicate that people who reported, on average, higher scores on one variable also reported, on average, higher scores on the other variable. Red edges represent negative (inhibitory) effects that indicate that people who reported, on average, higher scores on one variable also reported, on average, lower scores on the other variable. Edges with weights above 0.05 are plotted with a thickness corresponding to their magnitude. Note that between-person analyses provide interindividual associations and therefore directions of effects cannot be specified. **b** Standardized node strength, which represents the conditional association of a node with other nodes in the network.

Figure 4a shows between-person relations in the broad COVID-19 vaccines network (weights of edges discussed in the text are reported in parentheses). These between-person relations indicate associations between nodes at the population level (i.e., interindividual). At the between-person level, Intention vaccine was most strongly related to Vaccines attitude (0.51) and General attitude vaccination (0.37), indicating that at the population level these variables tend to co-occur, and these relations cannot be explained by any other variable in the network. Thus, people who reported, on average, higher intention to get vaccinated also reported, on average, a more positive attitude toward COVID-19 vaccines and vaccination in general, and vice versa. The network also showed a negative relation between vaccination intention (Intention vaccine) and negative affect related to COVID-19 vaccines (Vaccines negative affect; –0.18). This suggests that people who reported, on average, higher intention to get vaccinated also reported, on average, less negative emotions related to COVID-19 vaccines, and vice versa.

The standardized centrality measure “node strength” for the between-person broad COVID-19 vaccines networks is depicted in Fig. 4b (node strength values discussed in the text are reported in parentheses). The node Intention vaccine was relatively central in the network (0.46), indicating that it has associations with many other nodes. The nodes Vaccines attitude and Vaccines trust showed the relatively highest strength (1.61, 1.21, respectively). This indicates that these nodes have the highest conditional association with other nodes in the between-person broad COVID-19 vaccines network, thus these are highly connected to other nodes in this network.

This interindividual level analysis, however, provided no information on the direction of effects. The longitudinal design of this study also allowed for calculating within-person predictive effects between variables. This resulted in a temporal broad COVID-19 vaccines network with directed edges between nodes.

#### Temporal broad COVID-19 vaccines network

**Fig. 5:**
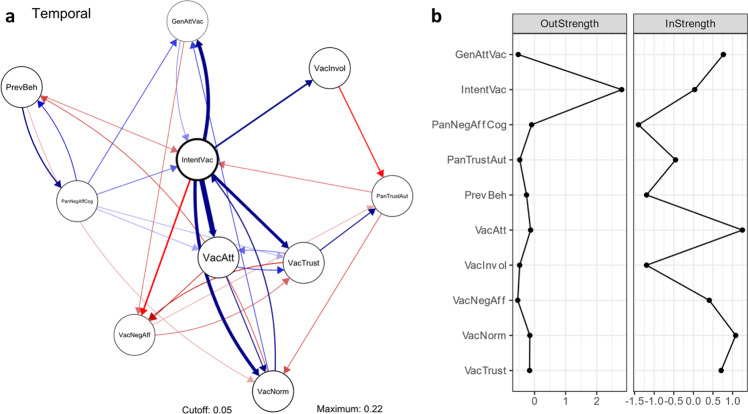
Temporal broad COVID-19 vaccines network. See Fig. 4 for node legend. **a** Network: nodes represent measured variables with border width indicating stability. Edges indicate predictive effects for the next measurement when controlling for every other node in the network. Edge weights, as indicated by width based on the correlation coefficient, represent the strength of effects (see Table 2). Blue edges indicate positive (excitatory) effects and red edges indicate negative (inhibitory) effects. Edges with weights above 0.05 are plotted with a thickness corresponding to their magnitude. **b** Standardized node strength, which represents direct effects of specific nodes in the network. InStrength refers to edges affecting that specific node, and OutStrength refers to edges originating from that node and affecting other nodes.

Figure 5a shows temporal effects in the broad COVID-19 vaccines network. These temporal effects indicate intraindividual predictive effects from one measurement to the next (after controlling for other nodes in the network), covering a timeframe of approximately three weeks in this study. A complete overview of the edge weights is provided in Table 2. Edges between nodes in temporal networks indicate either a causal effect, or an effect with an (unknown) underlying cause. Border width of nodes indicates autoregression (i.e., to what extent nodes are predicted by the same node in the previous measurement), with thicker node borders indicating higher node stability. The most stable variables in the temporal vaccines network were intention to get vaccinated against COVID-19 (Intention vaccine; 0.42), social norms on getting vaccinated (Vaccines social norm; 0.16), and attitudes toward COVID-19 vaccines (Vaccines attitude; 0.15).

**Table 2:** Complete overview of edge weights of (pruned) temporal COVID-19 vaccines network.

From node	To node									
	General attitude vaccination	Intention vaccine	Pandemic negative affect and cognitions	Pandemic trust in authorities	Preventive behaviors	Vaccines attitude	Vaccines involvement	Vaccines negative affect	Vaccines social norm	Vaccines trust
General attitude vaccination	**0.06**	0.02						−0.03		
Intention vaccine	0.16	**0.42**				0.22	0.09	−0.08	0.15	0.14
Pandemic negative affect and cognitions	0.04	0.03	**0.11**		0.05	0.02				0.02
Pandemic trust in authorities		−0.03		**0.11**					−0.03	
Preventive behaviors		−0.03	0.07		**0.14**				−0.02	
Vaccines attitude						**0.15**		−0.04	0.06	0.04
Vaccines involvement				−0.06			**0.14**			
Vaccines negative affect				−0.02				**0.10**		−0.03
Vaccines social norm	0.04	0.06			−0.04				**0.16**	
Vaccines trust				0.06		0.03		−0.05		**0.11**

Read rows (first column) as nodes from which the edge originates, and columns as the predicted nodes. Diagonal values indicate node stability.

Intention to get vaccinated showed to be predictive of every variable related to vaccination (i.e., General attitude vaccination, Vaccines attitude, Vaccines involvement, Vaccines negative affect, Vaccines social norm, and Vaccines trust). The strongest edges in the temporal vaccines network were observed from intention to get vaccinated against COVID-19 (Intention vaccine) to one’s attitude (i.e., emotions, beliefs, and behaviors) toward the vaccines (Vaccines attitude; 0.22) and vaccinations in general (General attitude vaccination; 0.16). This suggests that COVID-19 vaccination intention predicts how one feels, thinks, and acts regarding the vaccines and one’s general attitude regarding vaccination to prevent diseases.

Conversely, intention to get vaccinated against COVID-19 was predicted by few other nodes in the network. The relatively strongest predictor of intention to get vaccinated was social norms on getting vaccinated against COVID-19 (Vaccines social norm; 0.06). As mentioned, intention to get vaccinated against COVID-19 predicted social norms on getting vaccinated (Vaccines social norm; 0.15). Such bidirectional edges can be interpreted as potential feedback loops, indicating that when social norms on getting vaccinated against COVID-19 change, over time one’s intention to get vaccinated also changes, and vice versa.

The standardized centrality measure “strength” for the temporal COVID-19 vaccines network (depicted in Fig. 5b) confirmed that intention to get vaccinated predominately predicted other nodes and was hardly predicted by nodes in the network (OutStrength 2.80; InStrength 0.03). Results also showed a relatively high InStrength for attitude toward COVID-19 vaccines (Vaccines attitude; 1.25), social norms on getting vaccinated against COVID-19 (Vaccines social norm; 1.08), vaccinations in general (General attitude vaccination; 0.77), and trust in the science behind and in the developers of COVID-19 vaccines (Vaccines trust; 0.71). This indicates that scores on these nodes in the next measurement were predicted relatively well by other nodes in the network.

In summary, on a within-person level, few variables in the network predicted intention to get vaccinated against COVID-19, whereas every variable concerning vaccination was predicted by vaccination intention. Combining the within- and between-person levels of the network revealed that although attitudes toward COVID-19 vaccines are relatively central in the network and strongly related to intention to get vaccinated, such attitudes are not predictive of vaccination intention. Finally, the correlation between edges in the temporal and between-person networks is modest (*r* = 0.41, *z* = 0.43), suggesting a moderate relation between within-person predictive effects over time and average population-level associations between nodes. The contemporaneous broad COVID-19 vaccines network, encompassing undirected relations between variables within the same measurement, is provided in Supplementary Note 5.

#### Sensitivity analyses

Analyses described above were conducted for the default dataset, consisting of six measurements (T1–T6) in which missing values on vaccination intention from T5 onwards (for respondents who received a COVID-19 vaccine; *n* = 224) were imputed with their last available score from prior waves. Multiple sensitivity analyses were conducted to test the stability of the results. Analyses were rerun with other measurements (i.e., T1–T5 and T2–T6) and a different imputation method for missing values of respondents that indicated they were vaccinated (i.e., scores 6 and 7 representing high intention to get vaccinated). Finally, analyses were run with a sample in which respondents who missed one measurement in between the six measurements were added to the longitudinal sample after imputing their missing data. The missing scores were imputed from the wave prior to the wave a participant had missed. The main results of this study remained valid irrespective of these variations in the dataset: COVID-19 vaccination intention showed relatively strong predictive effects for other variables related to COVID-19 vaccines, but other variables in the network showed relatively weak predictive effects for vaccination intention, and vaccination intention was relatively stable over time.

Regarding variables predictive of vaccination intention, bidirectional predictive effects between social norms on getting vaccinated against COVID-19 and intention to get vaccinated were found in most variations of the dataset (i.e., T1–T6 and T2–T6, regardless of imputation method). Moreover, a predictive effect from Vaccines trust on COVID-19 vaccination intention was found in the T1–T5 variation of the dataset. The possibility of a small effect of that variable on (earlier) COVID-19 vaccination intention therefore cannot be excluded. Nevertheless, the main results of this study stand despite variations in included measurements and imputed missing values on vaccination intention for vaccinated respondents. A summary of the results from these sensitivity analyses is provided in Table 3 (see Supplementary Note 6 for detailed results).

**Table 3:** Summary of sensitivity analysis results.

Intent vaccine	General attitude vaccination	Intent vaccine	Pandemic negative affect and cognitions	Pandemic trust authorities	Preventive behaviors	Vaccine attitude	Vaccine involvement	Vaccine negative affect	Vaccine social norm	Vaccine trust
T1–T6: impute last known										
Ingoing	0.02	0.42	0.03	−0.03	−0.03				0.06	
Outgoing	0.16	0.42				0.22	0.09	−0.08	0.15	0.14
T1–T6: impute seven										
Ingoing		0.43		−0.03	−0.03				0.08	
Outgoing	0.16	0.43				0.23	0.08	−0.09	0.15	0.14
T1–T5: impute last known										
Ingoing	0.05	0.40								0.08
Outgoing	0.16	0.40				0.20	0.10	−0.07	0.10	0.16
T1–T5: impute seven										
Ingoing	0.05	0.41								0.07
Outgoing	0.16	0.41				0.20	0.10	−0.08	0.10	0.16
T2–T6: impute last known										
Ingoing		0.33		−0.05		0.03			0.04	
Outgoing	0.08	0.33				0.18	0.04	−0.05	0.11	0.10
T2–T6: impute seven										
Ingoing	−0.04	0.35		−0.05		0.02			0.06	
Outgoing	0.08	0.35				0.19		−0.06	0.11	0.10

Displays edge weights of edges to (ingoing) and from (outgoing) intention to get vaccinated against COVID-19 based on variations of the dataset in measurements and imputation method for missing values on COVID-19 vaccination intention from T5 onwards (for respondents who received a COVID-19 vaccine).

## Discussion

The current research examined the temporal dynamics of intention to get vaccinated against COVID-19 by conducting a longitudinal survey study during the phase of developing and enrolling the vaccines. Two main results were obtained from this study.

First, COVID-19 vaccination intention was a stronger predictor of other variables related to COVID-19 vaccines in the broad vaccines network than other variables in the network of vaccination intention. While cross-sectional results indicated that COVID-19 vaccination intention was relatively strongly related to attitudes toward the vaccines, results obtained from temporal analyses showed that these effects stem from vaccination intention. As such, intention to get vaccinated mainly predicted other vaccination-related variables included in the network, and predictive effects of other variables for vaccination intention were relatively weak and not robust. This implies that variables included in the broad COVID-19 vaccines network did not convincingly account for changes in vaccination intention. While these results are consistent with several studies showing relations between attitudes and vaccination intention^2,9,10,12,17^, they are less consistent with experimental research that points toward elements of attitudes toward COVID-19 vaccines as predictive of vaccination intention^11,20^. Also, in some theoretical models on attitudes and behavior, intention is generally seen as more of a consequence (and less of a cause) of the attitude, e.g.,^34^. In that light, the present findings are perhaps at odds with these models but fit well theories positing strong influences of behavior on attitudes, such as self-perception theory^35^ and cognitive dissonance theory^36^.

The somewhat puzzling result that vaccination intention is on the one hand only weakly predicted by the variables in the broad COVID-19 vaccines network and on the other hand has a strong impact on these variables might be explained by combining the network theory of attitudes^30,37^ with self-perception theory^35^. Self-perception theory holds that individuals often base their attitudes on behavior, which from a network perspective would imply that behavior is a central node in the attitude network. Combining this with the postulate of the network theory of attitudes that directing attention and thinking about attitude objects increases the connectivity of the attitude network has an interesting implication for the current study. Given the centrality of vaccines in the public discussion, one can expect that individuals’ attitude networks were strongly connected. In such strongly connected networks, an initial decision (i.e., the intent to get vaccinated) that might be based on rather random factors would be amplified and stabilized through the strong connectivity between nodes.

Another possible explanation for COVID-19 vaccination being more predictive than predicted lies in how committed people are to their intention. Research on cognitive dissonance demonstrated that merely committing to behavior can already result in attitude change^38^. COVID-19 vaccination intention might have become predictive of other elements in the network if many people committed to getting vaccinated early on. This is because a strong commitment to getting vaccinated, for instance, due to social norms, might cause dissonance that is resolved by attitude change. An implication of this would be that campaigns aimed at promoting vaccine uptake should be implemented at an early stage, preferably before the actual rollout of the vaccine.

To conclude, the first result indicates that COVID-19 vaccination intention is presumably difficult to change. This is substantiated by the finding that vaccination intention was relatively stable over time, which is in line with other research conducted during the same timeframe^39,40^.

As a second result, this study demonstrates the importance of a research design that differentiates effects on within- and between-person levels when studying vaccination intention. In line with other research, we observed a relation between attitudes and COVID-19 vaccination intention, but the within-person level showed that the direction of this effect goes to and not from vaccination intention. Therefore, when solely studying between-person effects, as with cross-sectional research, one would fail to identify the direction of predictive effects regarding vaccination intention. However, we also observed that attitudes toward COVID-19 vaccines showed a relatively strong (yet not predictive) relation with COVID-19 vaccination intention on both within- and between-person levels. This suggests that attitudes are important for understanding COVID-19 vaccination intention on an interindividual level. Therefore, when solely studying within-person effects, one would miss valuable insights that can contribute to understanding complex psychological systems at the population level. Such insights can foster the understanding of the interplay of variables of individuals compared to others regarding vaccination intention during pandemics. In summary, different yet complementary insights can be derived when studying vaccination intention from both within- and between-person levels.

The current study contributes to the existing literature on vaccine hesitancy such as the Vaccine Hesitancy Determinants Matrix as proposed by the SAGE Working Group on Vaccine Hesitancy^1^. This matrix classifies determinants as (1) individual and group influences, (2) contextual influences, and (3) vaccine-specific influences. Our research included determinants of the classifications of individual and group influences and vaccine-specific influences, in the broad COVID-19 vaccines networks. It also provided insight into determinants of contextual influences by including a timeline of media coverage throughout the course of the study. The current study contributes to this literature on vaccine hesitancy by demonstrating the importance of investigating directions of relations between determinants and vaccine hesitancy. Future research could build upon our research by including determinants of every group in the empirical networks, potentially even every determinant in the matrix, and investigating predictive effects between these determinants.

Finally, several limitations and recommendations should be addressed. While the current study expanded attitude networks to include a broader set of relevant variables, future research could aim to include more variables relevant to intention to get vaccinated with new vaccines developed during pandemics. Next, the survey administered in this study, although inspired by validated scales, was constructed for and tailored to COVID-19 vaccines and therefore not independently validated. Furthermore, although predictive effects in longitudinal data provide indications of causal relations, interventions should be studied to make stronger causal inferences^41^. Future research could focus on causal relations to further explain and predict COVID-19 vaccine uptake, for instance by intervening in social norms surrounding vaccination against COVID-19. Also, although sensitivity analyses indicated comparable global network structures between the longitudinal sample and other respondents, we cannot precisely predict how results would be affected by including participants who dropped out. However, since the aim is not to present representative node scores but to provide insight into relations between nodes, attrition is not considered a substantial issue for this study. Finally, measuring vaccination intention and related variables repeatedly might have a dissonance-enhancing effect on attitudes. This implies that reporting a change in intention to get vaccinated against COVID-19 might cause dissonance, which can in turn result in attitude change to alleviate dissonance.

In conclusion, this research demonstrates the challenge of stimulating the uptake of vaccines developed during pandemics. This implies that promoting vaccine uptake in future pandemics would benefit most from strategies aimed at preventing polarization regarding the newly developed vaccines. Future research could build upon this research by expanding the variables included in the broad COVID-19 vaccines networks and testing research interventions based on these networks.

## Methods

### Participants and design

This large-scale survey study was approved by the Ethics Review Board of the University of Amsterdam (2020-SP-12849) and not preregistered due to its explorative nature. The initial sample consisted of 1500 Dutch respondents from a research panel (Ipsos). These respondents were representative of the Dutch population in terms of age, gender, education, and residential area. Respondents completed a survey with variables related to COVID-19 vaccines six times over a period of five months. Successive waves contained both recurring respondents and additional respondents to meet a number of approximately 1500 respondents per wave. The current study is based on the recurring group of respondents, forming the longitudinal sample. There was no significant difference between the longitudinal sample and other respondents on global network measures (see Supplementary Note 7 for more information), indicating that the longitudinal sample is representative of the research population of each wave. We administered the survey on six occasions (approximately once every three weeks) to examine how attitudes develop over time. The final longitudinal sample that finished the survey in each of the six waves consisted of *N* = 744 (see Table 4). This sample size was expected to provide sufficient power, since it well exceeds the advised number of respondents required for accurate estimation of several network models with a moderate amount of nodes^33,42^. The survey contained two attention checks to ensure data quality. The attention checks stated: “To ensure that you maintain your attention during this survey, we ask that you select ‘Strongly agree’ [/‘Strongly disagree’] here”. Failing both attention checks stopped the survey automatically after the second wrong answer and thereby led to exclusion. The survey displayed a progress bar while participants filled in the survey to prompt completion.

**Table 4:** Specifications per wave leading up to the final longitudinal sample (*N* = 744).

Sample formation	Wave 1	Wave 2	Wave 3	Wave 4	Wave 5	Wave 6
Start data collection	December 8, 2020	January 6, 2021	February 3, 2021	March 3, 2021	March 31, 2021	April 28, 2021
End data collection	December 16, 2020	January 12, 2021	February 10, 2021	March 10, 2021	April 7, 2021	May 6, 2021
Valid *N*	1501	1132	959	866	799	744

### Measures

An expert meeting was organized for advice on which constructs to include in the survey. The final survey consisted of 35 items related to attitudes (see Supplementary Note 8 for the complete survey). Answers were provided on a 7-point Likert scale ranging from 1 (strongly disagree) to 7 (strongly agree). The first part (11 items) surveyed people’s perspectives on the COVID-19 pandemic, including the disease, and contained one item on general attitude toward vaccination. COVID-19-related items were inspired by previous research into the pandemic^18,43^. The second part (24 items) surveyed people’s perspectives on the COVID-19 vaccines. Items were inspired by validated vaccination uptake scales^44–46^, but adjusted to fit the unique COVID-19 context. Both parts consisted of items tapping into elements relevant to attitudes: affective items on emotions, cognitive items on beliefs, and finally items on behavior and behavioral intentions, including intention to get vaccinated against COVID-19. Respondents also indicated demographic variables, and additional information on health and COVID-19 infections.

The survey was largely similar across waves, with two exceptions. From wave 5 onwards, respondents were asked about their vaccination status, and the item on vaccination intention was skipped for respondents who reported to have received the COVID-19 vaccine. These missing values for vaccination intention (for 224 vaccinated respondents) were imputed with the respondent’s answer on that item in the prior wave. Different imputation such as the highest possible score had no effect on the main results (see paragraph sensitivity analyses in the Results section). Imputing the last known score resulted in more variance than imputing the highest score and was therefore chosen. Also from wave 5 onwards, the survey included an item covering an emerging public debate about granting different rights to individuals based on their vaccination status (see Supplementary Note 8 for more details). This item was not included in the network analyses, since the networks only contain measures that have scores on every measurement.

After collecting the data, items were combined to form nodes to include in the network (see section on data analysis). The resulting nodes are displayed in Table 5. The score of nodes that consisted of more than one item was calculated with mean scores of the items.

**Table 5:** Nodes (psychological variables) based on items in the survey, including item examples, number of items per node and reliability of nodes as observed in the current study (calculated with every respondent completing the survey in either of the six waves).

Node	Items per node, reliability	Examples of items per node (/in the same text line means separate item in survey)
Intention vaccine	1	I am getting vaccinated against COVID-19
Vaccines attitude	11, *a* = 0.95	I have a good feeling about COVID-19 vaccines/People who do not want to get vaccinated against COVID-19 make me angry/COVID-19 vaccines protect well against COVID-19/The side effects of COVID-19 vaccines have been sufficiently studied
Vaccines negative affect	3, *a* = 0.88	I worry about the safety of COVID-19 vaccines/I feel misled about the safety of COVID-19 vaccines
Vaccines involvement	3, *a* = 0.75	I follow the news about COVID-19 vaccines/I know much about COVID-19 vaccines
Vaccines social norm	2, *r*_*sb*_ = 0.90	I think my family and friends get vaccinated against COVID-19/I think everyone should get vaccinated against COVID-19
Vaccines trust	2, *r*_*sb*_ = 0.94	I trust the science behind COVID-19 vaccines/I trust the developers of COVID-19 vaccines
General attitude vaccination	1	I am in favor of using vaccinations to prevent disease
Pandemic negative affect and cognitions	6, *a* = 0.83	I am afraid of getting infected with the coronavirus/I worry about losing friends or family to COVID-19/COVID-19 is dangerous to my health
Pandemic trust authorities	1	I trust the authorities responsible for fighting the COVID-19 pandemic
Preventive behaviors	3, *a* = 0.74	I keep 1.5 meters away from others as much as possible/I wear a face mask in public areas

All items were answered on a scale ranging from 1 (Strongly disagree) to 7 (Strongly agree). Mean scores of items were calculated for nodes based on more than one item.

### Procedure

Participants became members of Ipsos’ research panel by signing up (opt-in panel). They are digitally invited to participate in this study. This invitation included a short description of the topic of the questionnaire. Participation is rewarded with credits to spend in online shops. The complete survey consisted of approximately 45 questions, including consent, attention checks, and demographics. The median time spend to complete it was approximately 5 min. The questionnaire was programmed with the online survey software Qualtrics. Participants’ answers were linked to Qualtrics through the Panel Company Integration method provided by Qualtrics.

Prior to starting the questionnaire, participants received information about participating in this study and provided consent on participation and data usage (see S8 for more details on instructions). Only participants that gave permission and finished the survey received invitations for subsequent waves.

After the sixth wave, two more waves were conducted that were not included in this study, with the seventh wave including interventions at the beginning of the survey. These interventions are not the focus of the current paper and will thus be presented elsewhere.

### Data analysis

The R code and data sets are made available (see Supplementary Note 9). As mentioned, survey items on attitudes toward the pandemic and vaccines were combined to form the nodes included in the broad COVID-19 network. A predetermined set of items was treated as either a single item node (e.g., vaccination intention and general attitude toward vaccination) or a combined set of items (e.g., trust and social norms). Remaining items were reduced to a concise number of components that describe the data with principal axis factoring. A detailed explanation of the construction of nodes is provided in Supplementary Note 10.

Networks were estimated with the *panelgvar* model (a graphical vector-autoregression model developed for panel data) in the package *psychonetrics* version 0.10^32,47^. We calculated both the saturated network model (i.e., model in which all edges are included) and the pruned network model in which edges that were not significant at *a* = 0.05 were fixed to zero to compare which model best fits the data observed in this study. This analysis provides three types of psychometric networks: a between-person network providing an overview of how variables relate at the population level and two networks with average within-person effects on the individual level, that is, temporal and contemporaneous networks^32^.

Edges in the between-person network represent relations between stable means and indicate which nodes are related at the interindividual level^32^. These edges can be interpreted as partial correlations, with the weight of edges indicating the strength of the relation. Edges in temporal networks indicate predictive effects between nodes. That is, the degree to which one node predicts another node in the next measurement when controlling for every other node in the network (i.e., conditional association over time between two nodes). These predictive effects are obtained by regressing each node on every other node, including itself, in the previous measurement (i.e., lag-1), and thus require repeated measurements. The calculated directed predictive effects from measurement *t*–1 to measurement *t* are partial correlations that are displayed in the network by edges with arrows. Edge weights indicate the size of the predictive effect and can be interpreted as (directed) partial correlations. Variance and covariance that cannot be explained by the modeled temporal effects provide input for contemporaneous networks: edges in these networks are calculated with the residuals of temporal networks. Contemporaneous edges can be interpreted as partial correlations between nodes in the same measurement after controlling for temporal effects (i.e., controlling for every other node in the same and previous measurement). Additional information on these types of networks is provided by Epskamp et al.^48^. To calculate correlations between edges in the different networks, the ingoing and outgoing edges between nodes in the temporal network were summed to one measure.

Furthermore, network analysis enables calculating node centrality measures that facilitate interpretation of these networks. “Strength” is among the most commonly used centrality measure in psychological networks^49^ and thus reported here. Node strength represents the conditional association between a node and other nodes in the network. This metric is calculated as the sum of the absolute edge weights of edges one node has with connected nodes. Directed edges in temporal networks distinguish between effects to and from a node, allowing us to distinguish two types of strength: InStrength (i.e., edges directed toward a specific node) and OutStrength (i.e., edges directed from a specific node to other nodes).

## Supplementary information

Supplementary Information

## Data Availability

Data with codebook are made available on OSF (https://osf.io/357h4/).

## Code availability

R code is made available on OSF (https://osf.io/357h4/).
