# Supplementary material for: Understanding change in COVID-19 vaccination intention with network analysis of longitudinal data from Dutch adults
Source: NPJ Vaccines. 2022 Oct 1;7:114. doi: 10.1038/s41541-022-00533-6 (PMC9526393; doi:10.1038/s41541-022-00533-6)
Supplement: Supplementary file 1 — Supplementary Information [file 41541_2022_533_MOESM1_ESM.docx]

Supplementary Information

Manuscript ‘Understanding change in COVID-19 vaccination intention with network analysis of longitudinal data from Dutch adults’

Contents

[Supplementary Note 0: Timeline data 2](#_Toc111112125)

[Supplementary Note 1: Node-specific descriptive statistics for each measurement 3](#_Toc111112126)

[Supplementary Note 2: Change in COVID-19 vaccination intention scores 4](#_Toc111112127)

[Supplementary Note 3: *Panelgvar* model fit statistics 6](#_Toc111112128)

[Supplementary Note 4: Edge weights with confidence intervals and node strength 7](#_Toc111112129)

[Supplementary Note 5: Contemporaneous broad COVID-19 vaccines network 16](#_Toc111112130)

[Supplementary Note 6: Sensitivity analyses 18](#_Toc111112131)

[Supplementary Note 7: Comparing longitudinal sample with other respondents 19](#_Toc111112132)

[Supplementary Note 8: Survey items and instructions 20](#_Toc111112133)

[Supplementary Note 9: R code and data 22](#_Toc111112134)

[Supplementary Note 10: Node construction with items per node 24](#_Toc111112135)

[References 29](#_Toc111112136)

## Supplementary Note 0: Timeline data

The data underlying the Dutch timeline is provided on OSF (see .xlsx file): [https://osf.io/357h4/](https://osf.io/357h4/?view_only=a386a5e1288f435fabeba4aa8fbc5cb4)

## Supplementary Note 1: Node-specific descriptive statistics for each measurement

Supplementary Table 1 – Descriptive statistics for each node in the COVID-19 vaccines networks.

|  | T1 |  | T2 |  | T3 |  | T4 |  | T5 |  | T6 |  |
| --- | --- | --- | --- | --- | --- | --- | --- | --- | --- | --- | --- | --- |
|  | *M* | *SD* | *M* | *SD* | *M* | *SD* | *M* | *SD* | *M* | *SD* | *M* | *SD* |
| General Attitude Vaccination | 6.03 | 1.37 | 6.21 | 1.34 | 6.23 | 1.33 | 6.20 | 1.32 | 6.16 | 1.42 | 6.27 | 1.29 |
| Intention Vaccine | 5.43 | 1.92 | 5.93 | 1.75 | 5.97 | 1.74 | 5.98 | 1.77 | 6.00 | 1.77 | 6.08 | 1.69 |
| Pandemic Negative Affect and Cognitions | 4.87 | 1.14 | 4.92 | 1.12 | 4.84 | 1.14 | 4.76 | 1.16 | 4.78 | 1.16 | 4.73 | 1.18 |
| Pandemic Trust in Authorities | 5.19 | 1.49 | 5.24 | 1.51 | 5.01 | 1.59 | 5.04 | 1.55 | 4.87 | 1.61 | 4.89 | 1.63 |
| Preventive Behaviors | 6.24 | 0.93 | 6.25 | 0.93 | 6.20 | 0.95 | 6.15 | 1.05 | 6.12 | 1.06 | 6.10 | 1.04 |
| Vaccines Attitude | 4.68 | 1.41 | 5.03 | 1.39 | 4.98 | 1.39 | 4.91 | 1.43 | 4.88 | 1.42 | 4.96 | 1.44 |
| Vaccines Involvement | 5.01 | 1.19 | 5.23 | 1.12 | 5.24 | 1.16 | 5.14 | 1.16 | 5.14 | 1.18 | 5.14 | 1.18 |
| Vaccines Negative Affect | 3.63 | 1.63 | 3.10 | 1.69 | 3.25 | 1.67 | 3.21 | 1.67 | 3.52 | 1.73 | 3.35 | 1.72 |
| Vaccines Social Norm | 5.08 | 1.44 | 5.52 | 1.34 | 5.55 | 1.39 | 5.51 | 1.41 | 5.53 | 1.40 | 5.59 | 1.39 |
| Vaccines Trust | 5.09 | 1.54 | 5.43 | 1.52 | 5.30 | 1.49 | 5.25 | 1.54 | 5.20 | 1.54 | 5.17 | 1.55 |

## Supplementary Note 2: Change in COVID-19 vaccination intention scores

Supplementary Table 2.1 – Percentage of respondents in the full sample and divided based on gender that changed their score on the 7-point Likert answer scale for COVID-19 vaccination intention for each wave. Percentages are calculated per row with the number of respondents per category divided by total per wave.

| Change in score | -6 | -5 | -4 | -3 | -2 | -1 | 0 | 1 | 2 | 3 | 4 | 5 | 6 |
| --- | --- | --- | --- | --- | --- | --- | --- | --- | --- | --- | --- | --- | --- |
| **Full Sample** | | | | | | | | | | | | | |
| T1 -> T2 | 0% | 0% | 0% | 0.4 % | 1.21 % | 3.9 % | 58.47 % | 22.18 % | 8.87 % | 3.23 % | 1.48 % | 0.13 % | 0.13 % |
| T2 -> T3 | 0% | 0.13 % | 0.13 % | 0.94 % | 1.75 % | 8.74 % | 73.52 % | 10.48 % | 2.96 % | 1.21 % | 0% | 0.13 % | 0% |
| T3 -> T4 | 0% | 0.13 % | 0.27 % | 0.81 % | 1.48 % | 8.6 % | 76.61 % | 9.41 % | 1.88 % | 0.54 % | 0% | 0.27 % | 0% |
| T4 -> T5 | 0% | 0.13 % | 0.13 % | 0.54 % | 1.61 % | 8.47 % | 76.75 % | 8.6 % | 2.96 % | 0.67 % | 0.13 % | 0% | 0% |
| T5 -> T6 | 0% | 0% | 0% | 0.27 % | 1.61 % | 4.97 % | 80.51 % | 8.74 % | 2.82 % | 0.54 % | 0.13 % | 0.13 % | 0.13 % |
| **Male** | | | | | | | | | | | | | |
| T1 -> T2 | 0% | 0% | 0% | 0% | 0.56 % | 4.44 % | 63.06 % | 18.89 % | 8.61 % | 3.06 % | 1.11 % | 0.28 % | 0% |
| T2 -> T3 | 0% | 0% | 0.28 % | 0.83 % | 1.94 % | 8.06 % | 77.22 % | 8.89 % | 1.11 % | 1.39 % | 0% | 0.28 % | 0% |
| T3 -> T4 | 0% | 0.28 % | 0.28 % | 0.28 % | 0.83 % | 6.67 % | 80% | 9.17 % | 1.67 % | 0.28 % | 0% | 0.56 % | 0% |
| T4 -> T5 | 0% | 0% | 0% | 0.28 % | 1.39 % | 8.33 % | 81.67 % | 5.56 % | 1.94 % | 0.56 % | 0.28 % | 0% | 0% |
| T5 -> T6 | 0% | 0% | 0% | 0.28 % | 1.11 % | 3.33 % | 82.5 % | 8.33 % | 3.61 % | 0% | 0.28 % | 0.28 % | 0.28 % |
| **Female** | | | | | | | | | | | | | |
| T1 -> T2 | 0% | 0% | 0% | 0.78 % | 1.82 % | 3.39 % | 54.17 % | 25.26 % | 9.11 % | 3.39 % | 1.82 % | 0% | 0.26 % |
| T2 -> T3 | 0% | 0.26 % | 0% | 1.04 % | 1.56 % | 9.38 % | 70.05 % | 11.98 % | 4.69 % | 1.04 % | 0% | 0% | 0% |
| T3 -> T4 | 0% | 0% | 0.26 % | 1.3 % | 2.08 % | 10.42 % | 73.44 % | 9.64 % | 2.08 % | 0.78 % | 0% | 0% | 0% |
| T4 -> T5 | 0% | 0.26 % | 0.26 % | 0.78 % | 1.82 % | 8.59 % | 72.14 % | 11.46 % | 3.91 % | 0.78 % | 0% | 0% | 0% |
| T5 -> T6 | 0.26 % | 0% | 0% | 0.26 % | 2.08 % | 6.51 % | 78.65 % | 9.11 % | 2.08 % | 1.04 % | 0% | 0% | 0% |

Supplementary Table 2.2 - Percentage of respondents in the full sample and divided based on age (younger or older than median age) that changed their score on the 7-point Likert answer scale for COVID-19 vaccination intention for each wave. Percentages are calculated per row with the number of respondents per category divided by total per wave.

| Change in score | -6 | -5 | -4 | -3 | -2 | -1 | 0 | 1 | 2 | 3 | 4 | 5 | 6 |
| --- | --- | --- | --- | --- | --- | --- | --- | --- | --- | --- | --- | --- | --- |
| **Full Sample** | | | | | | | | | | | | | |
| T1 -> T2 | 0% | 0% | 0% | 0.4 % | 1.21 % | 3.9 % | 58.47 % | 22.18 % | 8.87 % | 3.23 % | 1.48 % | 0.13 % | 0.13 % |
| T2 -> T3 | 0% | 0.13 % | 0.13 % | 0.94 % | 1.75 % | 8.74 % | 73.52 % | 10.48 % | 2.96 % | 1.21 % | 0% | 0.13 % | 0% |
| T3 -> T4 | 0% | 0.13 % | 0.27 % | 0.81 % | 1.48 % | 8.6 % | 76.61 % | 9.41 % | 1.88 % | 0.54 % | 0% | 0.27 % | 0% |
| T4 -> T5 | 0% | 0.13 % | 0.13 % | 0.54 % | 1.61 % | 8.47 % | 76.75 % | 8.6 % | 2.96 % | 0.67 % | 0.13 % | 0% | 0% |
| T5 -> T6 | 0% | 0% | 0% | 0.27 % | 1.61 % | 4.97 % | 80.51 % | 8.74 % | 2.82 % | 0.54 % | 0.13 % | 0.13 % | 0.13 % |
| **Age below Median** | | | | | | | | | | | | | |
| T1 -> T2 | 0% | 0% | 0% | 0.57 % | 1.7 % | 5.1 % | 52.97 % | 22.95 % | 11.9 % | 2.55 % | 1.7 % | 0.28 % | 0.28 % |
| T2 -> T3 | 0% | 0.28 % | 0.28 % | 0.85 % | 1.98 % | 11.05 % | 67.42 % | 12.75 % | 4.25 % | 1.13 % | 0% | 0% | 0% |
| T3 -> T4 | 0% | 0% | 0.28 % | 1.13 % | 1.98 % | 11.61 % | 72.52 % | 9.07 % | 2.55 % | 0.28 % | 0% | 0.57 % | 0% |
| T4 -> T5 | 0% | 0.28 % | 0% | 0.85 % | 1.7 % | 10.48 % | 72.52 % | 9.63 % | 3.4 % | 1.13 % | 0% | 0% | 0% |
| T5 -> T6 | 0% | 0% | 0% | 0.28 % | 2.83 % | 7.65 % | 72.52 % | 11.61 % | 4.25 % | 0.85 % | 0% | 0% | 0% |
| **Age above Median** | | | | | | | | | | | | | |
| T1 -> T2 | 0% | 0% | 0% | 0.26 % | 0.77 % | 2.81 % | 63.43 % | 21.48 % | 6.14 % | 3.84 % | 1.28 % | 0% | 0% |
| T2 -> T3 | 0% | 0% | 0% | 1.02 % | 1.53 % | 6.65 % | 79.03 % | 8.44 % | 1.79 % | 1.28 % | 0% | 0.26 % | 0% |
| T3 -> T4 | 0% | 0% | 0.26 % | 0.51 % | 1.02 % | 5.88 % | 80.31 % | 9.72 % | 1.28 % | 0.77 % | 0% | 0% | 0% |
| T4 -> T5 | 0% | 0% | 0.26 % | 0.26 % | 1.53 % | 6.65 % | 80.56 % | 7.67 % | 2.56 % | 0.26 % | 0.26 % | 0% | 0% |
| T5 -> T6 | 0.26 % | 0% | 0% | 0.26 % | 0.51 % | 2.56 % | 87.72 % | 6.14 % | 1.53 % | 0.26 % | 0.26 % | 0.26 % | 0.26 % |

## Supplementary Note 3: *Panelgvar* model fit statistics

BIC Saturated model = 94903.92

BIC Pruned model = 94426.45

Supplementary Table 3 - Model fit statistics from the pruned model

| Measure | Value |
| --- | --- |
| logl | -46806.58 |
| unrestricted.logl | -44621.61 |
| baseline.logl | -77699.25 |
| nvar | 60.00 |
| nobs | 1890.00 |
| npar | 123.00 |
| df | 1767.00 |
| objective | 15.55 |
| chisq | 4369.94 |
| pvalue | 0.00 |
| baseline.chisq | 66155.28 |
| baseline.df | 1770.00 |
| baseline.pvalue | 0.00 |
| nfi | 0.93 |
| pnfi | 0.93 |
| tli | 0.96 |
| nnfi | 0.96 |
| rfi | 0.93 |
| ifi | 0.96 |
| rni | 0.96 |
| cfi | 0.96 |
| rmsea | 0.04 |
| rmsea.ci.lower | 0.04 |
| rmsea.ci.upper | 0.05 |
| rmsea.pvalue | 1.00 |
| aic.ll | 93859.16 |
| aic.ll2 | 93908.36 |
| aic.x | 835.94 |
| aic.x2 | 4615.94 |
| bic | 94426.45 |
| bic2 | 94035.87 |
| ebic.25 | 94930.05 |
| ebic.5 | 95433.65 |
| ebic.75 | 95836.54 |
| ebic1 | 96440.86 |

## Supplementary Note 4: Edge weights with confidence intervals and node strength

**Between-person COVID-19 vaccines network**

Supplementary Table 4.1 - Edge weight table of between-person COVID-19 vaccines network

|  | General Attitude Vaccination | Intention Vaccine | Pandemic Negative Affect and Cognitions | Pandemic Trust Authorities | Preventive Behaviors | Vaccines Attitude | Vaccines Involvement | Vaccines Negative Affect | Vaccines Social Norm | Vaccines Trust |
| --- | --- | --- | --- | --- | --- | --- | --- | --- | --- | --- |
| General Attitude Vaccination |  | .37 |  |  |  |  |  |  |  | .18 |
| Intention Vaccine | .37 |  |  |  | .16 | .51 | -.10 | -.18 |  |  |
| Pandemic Negative Affect and Cognitions |  |  |  | .14 | .29 | .27 | .27 | .41 |  | -.06 |
| Pandemic Trust Authorities |  |  | .14 |  | .16 | -.13 |  |  |  | .48 |
| Preventive Behaviors |  | .16 | .29 | .16 |  |  | .10 | .11 |  |  |
| Vaccines Attitude |  | .51 | .27 | -.13 |  |  | .12 | -.14 | .32 | .39 |
| Vaccines Involvement |  | -.10 | .27 |  | .10 | .12 |  |  |  | .12 |
| Vaccines Negative Affect |  | -.18 | .41 |  | .11 | -.14 |  |  |  | -.32 |
| Vaccines Social Norm |  |  |  |  |  | .32 |  |  |  | .12 |
| Vaccines Trust | .18 |  | -.06 | .48 |  | .39 | .12 | -.32 | .12 |  |

Supplementary Figure 4.1 - Edge accuracy (confidence interval) between-person COVID-19 vaccines network


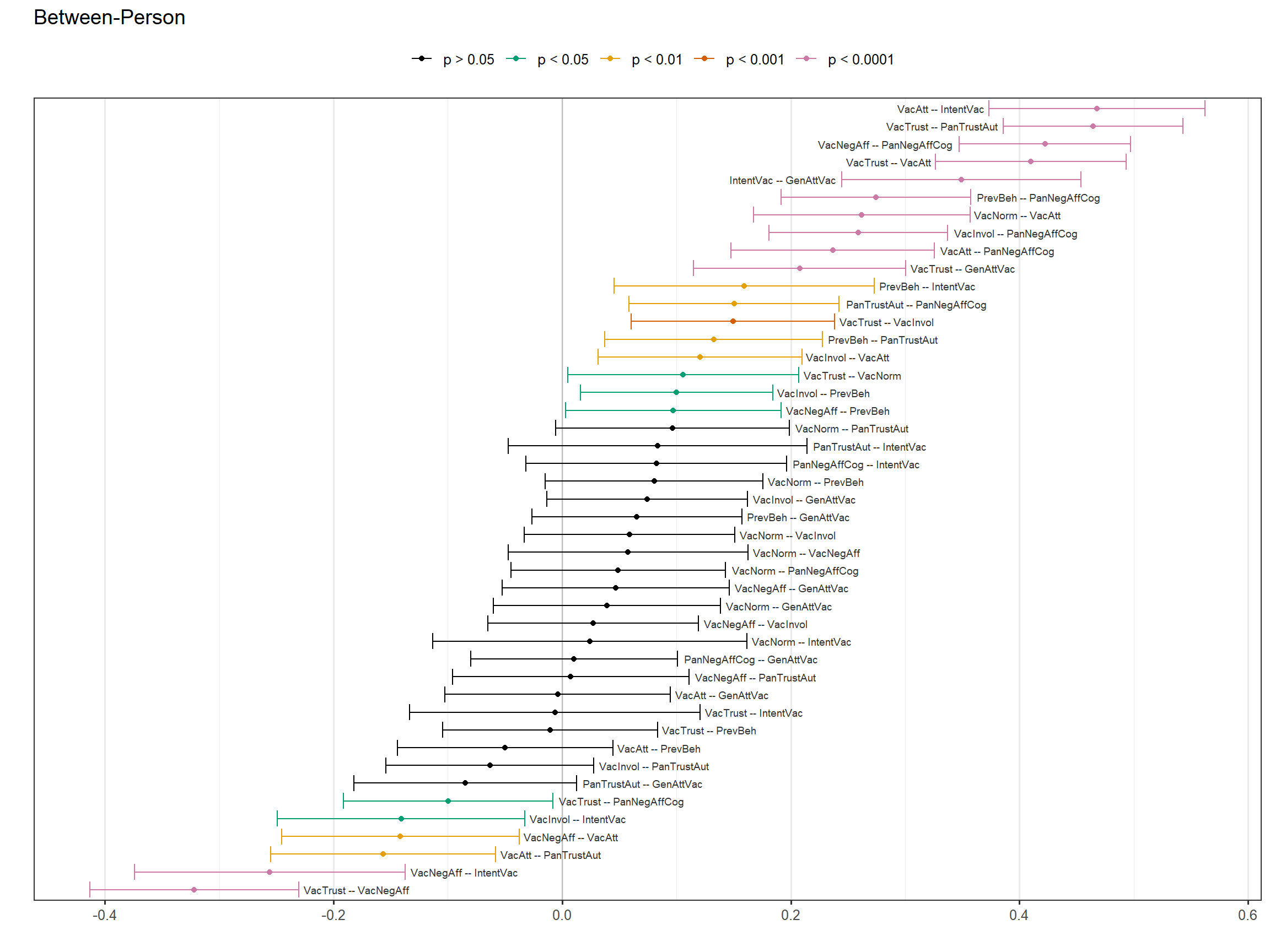


Note. The figure contains edges from the saturated model, whereas edges in the between-person COVID-19 vaccines network in the manuscript are based on the pruned model.

Supplementary Table 4.2 – Node strength table of between-person COVID-19 vaccines network

|  | Strength |
| --- | --- |
| General Attitude Vaccination | -1.11 |
| Intention Vaccine | 0.46 |
| Pandemic Negative Affect and Cognitions | 0.75 |
| Pandemic Trust in Authorities | -0.37 |
| Preventive Behaviors | -0.56 |
| Vaccines Attitude | 1.61 |
| Vaccines Involvement | -0.79 |
| Vaccines Negative Affect | 0.14 |
| Vaccines Social Norm | -1.35 |
| Vaccines Trust | 1.21 |

**Temporal COVID-19 vaccines network**

Supplementary Table 4.3 - Edge weight table of temporal COVID-19 vaccines network. Read rows (first column) as node from which the edge originates.

| To node  From node | General Attitude Vaccination | Intention Vaccine | Pandemic Negative Affect and Cognitions | Pandemic Trust in Authorities | Preventive Behaviors | Vaccines Attitude | Vaccines Involvement | Vaccines Negative Affect | Vaccines Social Norm | Vaccines Trust |
| --- | --- | --- | --- | --- | --- | --- | --- | --- | --- | --- |
| General Attitude Vaccination | **.06** | .02 |  |  |  |  |  | -.03 |  |  |
| Intention Vaccine | .16 | **.42** |  |  |  | .22 | .09 | -.08 | .15 | .14 |
| Pandemic Negative Affect and Cognitions | .04 | .03 | **.11** |  | .05 | .02 |  |  |  | .02 |
| Pandemic Trust in Authorities |  | -.03 |  | **.11** |  |  |  |  | -.03 |  |
| Preventive Behaviors |  | -.03 | .07 |  | **.14** |  |  |  | -.02 |  |
| Vaccines Attitude |  |  |  |  |  | **.15** |  | -.04 | .06 | .04 |
| Vaccines Involvement |  |  |  | -.06 |  |  | **.14** |  |  |  |
| Vaccines Negative Affect |  |  |  | -.02 |  |  |  | **.10** |  | -.03 |
| Vaccines Social Norm | .04 | .06 |  |  | -.04 |  |  |  | **.16** |  |
| Vaccines Trust |  |  |  | .06 |  | .03 |  | -.05 |  | **.11** |

Supplementary Figure 4.2 - Edge accuracy (bootstrap confidence intervals of edge weights) temporal COVID-19 vaccines network

*
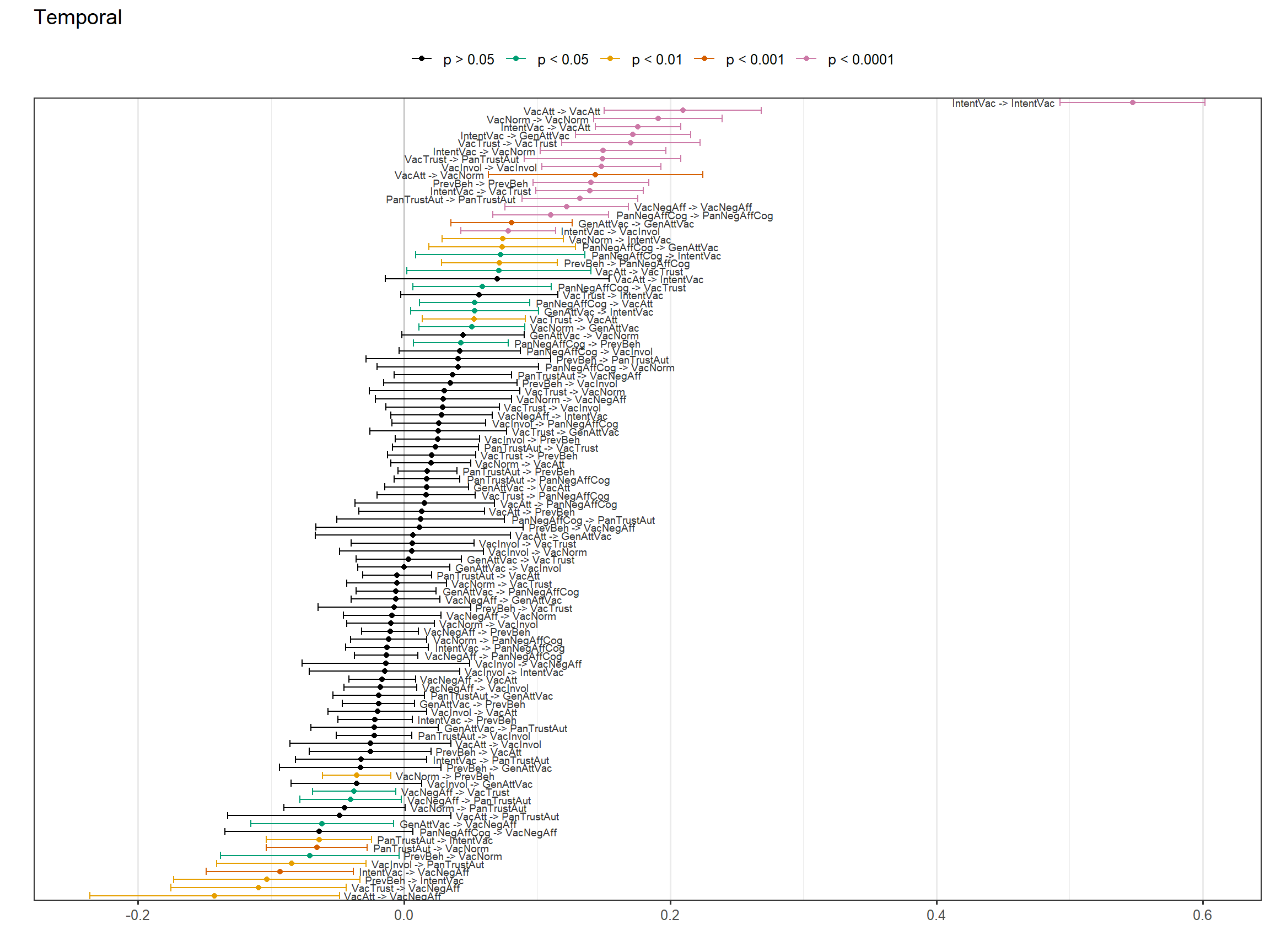
*

Note. Edges in the temporal COVID-19 vaccines network presented in the manuscript represent partial direct correlations (PDC), which are standardized beta values. The figure presented here contains (unstandardized) beta values and their confidence intervals of edges in the temporal network. Also, the figure contains edges from the saturated model, whereas edges in the temporal COVID-19 vaccines network in the manuscript are based on the pruned model.

Supplementary Table 4.4- Node strength table of temporal COVID-19 vaccines network

|  | OutStrength | InStrength |
| --- | --- | --- |
| General Attitude Vaccination | -0.52 | 0.77 |
| Intention Vaccine | 2.80 | 0.03 |
| Pandemic Negative Affect and Cognitions | -0.09 | -1.40 |
| Pandemic Trust in Authorities | -0.48 | -0.46 |
| Preventive Behaviors | -0.26 | -1.19 |
| Vaccines Attitude | -0.13 | 1.25 |
| Vaccines Involvement | -0.48 | -1.19 |
| Vaccines Negative Affect | -0.54 | 0.41 |
| Vaccines Social Norm | -0.15 | 1.08 |
| Vaccines Trust | -0.16 | 0.71 |

**Contemporaneous COVID-19 vaccines network**

Supplementary Table 4.5- Edge weight table of contemporaneous COVID-19 vaccines network

|  | General Attitude Vaccination | Intention Vaccine | Pandemic Negative Affect and Cognitions | Pandemic Trust Authorities | Preventive Behaviors | Vaccines Attitude | Vaccines Involvement | Vaccines Negative Affect | Vaccines Social Norm | Vaccines Trust |
| --- | --- | --- | --- | --- | --- | --- | --- | --- | --- | --- |
| General Attitude Vaccination |  | .23 | .08 |  | .09 | .09 |  |  | .03 | .06 |
| Intention Vaccine | .23 |  |  |  | -.02 | .32 |  | -.08 | .18 | .06 |
| Pandemic Negative Affect and Cognitions | .08 |  |  | .10 | .16 | .10 | .04 | .08 |  |  |
| Pandemic Trust Authorities |  |  | .10 |  | .04 | .06 |  |  |  | .15 |
| Preventive Behaviors | .09 | -.02 | .16 | .04 |  |  |  |  |  | .08 |
| Vaccines Attitude | .09 | .32 | .10 | .06 |  |  | .12 | -.19 | .20 | .30 |
| Vaccines Involvement |  |  | .04 |  |  | .12 |  | .08 | .10 | .16 |
| Vaccines Negative Affect |  | -.08 | .08 |  |  | -.19 | .08 |  |  | -.21 |
| Vaccines Social Norm | .03 | .18 |  |  |  | .20 | .10 |  |  | .05 |
| Vaccines Trust | .06 | .06 |  | .15 | .08 | .30 | .16 | -.21 | .05 |  |

Supplementary Figure 4.3 - Edge accuracy (bootstrap confidence interval of edge weights) contemporaneous COVID-19 vaccines network

*
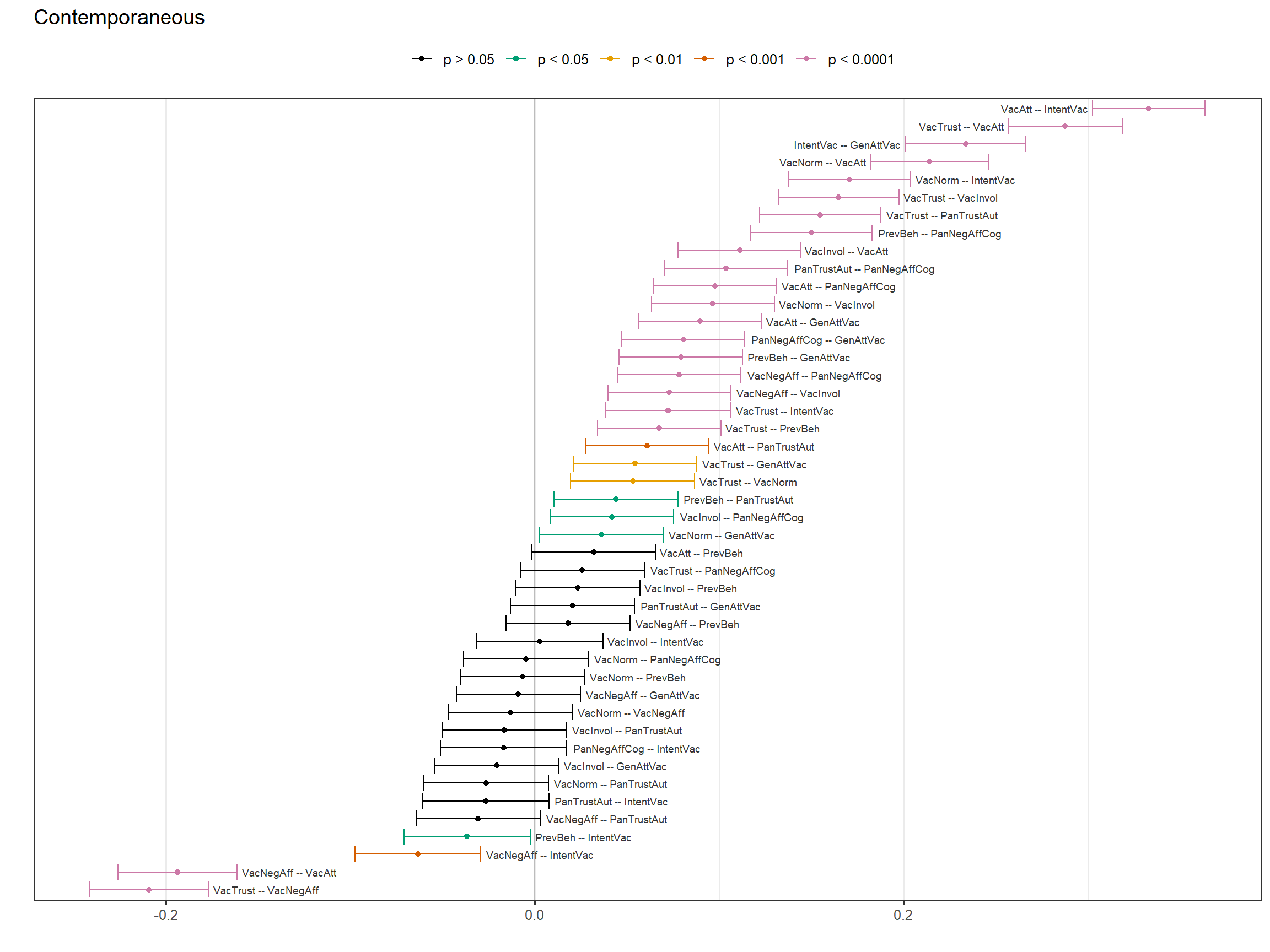
*

Note. The figure contains edges from the saturated model, whereas edges in the contemporaneous COVID-19 vaccines network in the manuscript are based on the pruned model.

Supplementary Table 4.6- Node strength table of contemporaneous COVID-19 vaccines network

|  | Strength |
| --- | --- |
| General Attitude Vaccination | -0.36 |
| Intention Vaccine | 0.63 |
| Pandemic Negative Affect and Cognitions | -0.42 |
| Pandemic Trust in Authorities | -1.03 |
| Preventive Behaviors | -0.93 |
| Vaccines Attitude | 2.12 |
| Vaccines Involvement | -0.63 |
| Vaccines Negative Affect | -0.14 |
| Vaccines Social Norm | -0.39 |
| Vaccines Trust | 1.14 |

## Supplementary Note 5: Contemporaneous broad COVID-19 vaccines network

Supplementary Figure 5a shows the contemporaneous relations in the broad COVID-19 vaccines network. These partial correlations, displayed as edges without arrows in the network, represent undirected associations between nodes. A complete overview of the edge weights in these networks is provided in Supplementary Note 4.

At the contemporaneous level, intention to get vaccinated against COVID-19 (*Intention Vaccine)* was positively related to *Vaccines Attitude* (.32), *General Attitude Vaccination* (.23), *Vaccines Social Norm* (.18) and *Vaccines Trust* (.06). This indicates that when one reports higher intention to get vaccinated, one is likely to report within the same measurement a more positive attitude toward COVID-19 vaccines and vaccination in general, a stronger social norm on getting vaccinated against COVID-19, and more trust in the science behind and in the developers of COVID-19 vaccines. *Intention Vaccine* was also negatively related to *Vaccines Negative Affect* (-.08), indicating that reporting higher intention to get vaccinated against COVID-19 tends to co-occur with reporting less negative emotions surrounding COVID-19 vaccines within the same measurement.

Results showed a moderate to strong correlation between edges in the temporal and contemporaneous COVID-19 vaccines networks (*r* = 0.63, *z* = 0.74). This implies that although there is a relation between predictive effects over time (i.e., between measurements) and associations within a survey (i.e., during measurements), there are notable differences between these networks. For instance, the temporal network showed that vaccination intention is predictive of involvement in COVID-19 vaccines (*Vaccines Involvement*; .09) in the next measurement, but the contemporaneous network did not indicate that these variables tend to co-occur within the same measurement. Furthermore, edges in the contemporaneous COVID-19 vaccines network showed a high correlation with the between-person network (*r* = .76, *z* = .99), suggesting a strong relation between within-person associations between nodes during measurements and average population level associations between nodes.

The standardized centrality measure ‘strength’ for the contemporaneous COVID-19 vaccines networks is depicted in Supplementary Figure 5b. The nodes Vaccines Attitude and Vaccines Trust show the relatively highest strength (2.12, 1.14, respectively). This indicates that these nodes have the highest average conditional association with other nodes in the contemporaneous broad COVID-19 vaccines network, which implies that these nodes are relatively important for this network.

Supplementary Figure 5 - Contemporaneous broad COVID-19 vaccines network. See Fig. 4 in the manuscript for interpretation guidelines. **a** Network: Positive (negative) edges indicate that people who reported higher scores on one variable also reported higher (lower) scores on the other variable. **b** Standardized node strength.
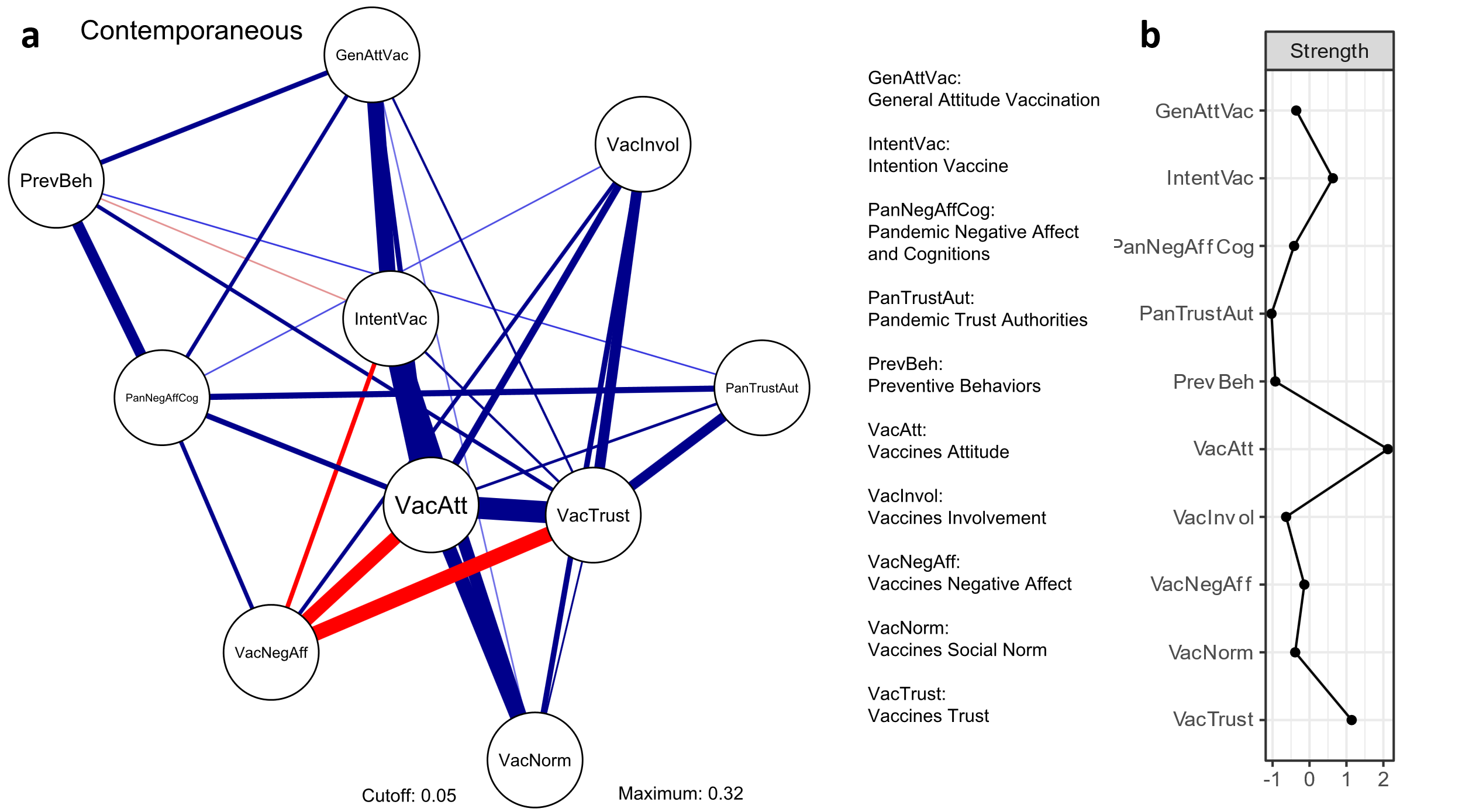


## Supplementary Note 6: Sensitivity analyses

Detailed results from the sensitivity analyses are provided on OSF (see .html file): https://osf.io/357h4/

## Supplementary Note 7: Comparing longitudinal sample with other respondents

The *longitudinal* sample was defined as every respondent present at all measurements (see Supplementary Table 7.1 for number of respondents and attrition rate per measurement). The *remaining* sample was defined by all other participants than the longitudinal sample present at each respective timepoint (remaining sample = total sample of approximately 1500 respondents per measurement – longitudinal sample). Supplementary Table 7.2 presents the results of the network comparison test NCT; version 2.2.1; ^1,2^ between the longitudinal and remaining samples. The NCT indicated that estimated cross-sectional networks did not display global statistical differences when examined at each timepoint. From Wave 1 to Wave 6, no significant differences were found between both samples with respect to network invariance (*M* = .06 - .11; *p* = .46 - .99), a measure for the distribution of edge weights, and global strength invariance (*S* = .09 - .23; *p* = .15 - .67), a measure for the global level of connectivity. This indicates that the total number of associations between nodes, and the estimated edge weight strengths, did not differ significantly between the longitudinal and remaining samples. Given global invariance, the local differences between both samples at each timepoint will not be discussed.

Supplementary Table 7.1 – Number of respondents for each measurement, including attrition rate

|  | Wave 1 | Wave 2 | Wave 3 | Wave 4 | Wave 5 | Wave 6 |
| --- | --- | --- | --- | --- | --- | --- |
| Total *N* Wave | 1501 | 1505 | 1502 | 1501 | 1506 | 1536 |
| Cumulative *N* | 1501 | 3006 | 4508 | 6009 | 7515 | 9051 |
| Longitudinal *N* | 1501 | 1132 | 959 | 866 | 799 | 744 |
| Attrition Rate (%) |  | -24.6% | -15.3% | -9.7% | -7.7% | -6.9% |

Supplementary Table 7.2 – Network comparison test (NCT) between longitudinal and remaining sample at each timepoint

|  | Network Invariance (*M*) | *p* | Global Strength Invariance (*S*) | *p* |
| --- | --- | --- | --- | --- |
| Wave 1 | 0.11 | .50 | 0.09 | .67 |
| Wave 2 | 0.08 | .89 | 0.17 | .23 |
| Wave 3 | 0.06 | .99 | 0.13 | .47 |
| Wave 4 | 0.11 | .46 | 0.23 | .15 |
| Wave 5 | 0.11 | .49 | 0.22 | .33 |
| Wave 6 | 0.10 | .52 | 0.13 | .56 |

## Supplementary Note 8: Survey items and instructions

Supplementary Table 8 – Items included in the survey

| Category | Item |
| --- | --- |
| Pandemic_Affect | I am afraid of getting infected with the corona virus. |
|  | I worry about losing friends or family to COVID-19. |
|  | I worry about the impact the COVID-19 pandemic will have on society. |
| Pandemic_Cognition | COVID-19 is dangerous to my health. |
|  | COVID-19 is much more dangerous than the flu. |
|  | It is likely that I will get COVID-19. |
| Pandemic_Behavior | I keep 1.5 meters away from others as much as possible. |
|  | I wash my hands often with soap and water. |
|  | I wear a face mask in public areas. |
| Pandemic_Trust | I trust the authorities responsible for fighting the COVID-19 pandemic. |
| General attitude vaccination | I am in favor of using vaccinations to prevent disease. |
| Vaccine_Affect | I worry about the safety of COVID-19 vaccines. |
|  | I am hopeful about COVID-19 vaccines. |
|  | I have mixed feelings about COVID-19 vaccines. |
|  | I have a good feeling about COVID-19 vaccines. |
|  | I am afraid of the injection when vaccinated with COVID-19 vaccines. |
|  | I feel misled about the safety of COVID-19 vaccines. |
|  | People who do not want to get vaccinated against COVID-19 make me angry. |
| Vaccine_Cognition | COVID-19 vaccines protect well against COVID-19. |
|  | To stop the pandemic, it is important that most people get vaccinated against COVID-19. |
|  | COVID-19 vaccines are safe for one's health. |
|  | The side effects of COVID-19 vaccines have been sufficiently studied. |
|  | People without a COVID-19 vaccination should no longer be allowed everywhere. |
|  | By getting vaccinated against COVID-19, I am protecting others from COVID-19. |
| Vaccine_Behavior* | I am getting vaccinated against COVID-19. |
|  | I encourage people to get vaccinated against COVID-19. |
|  | I avoid people who do not get vaccinated against COVID-19. |
|  | My religion or worldview has a lot of influence on my choice about vaccinating against COVID-19. |
| Vaccine_Trust | I trust the science behind COVID-19 vaccines. |
|  | I trust the developers of COVID-19 vaccines. |
| Vaccine_Norm | I think my family and friends get vaccinated against COVID-19. |
|  | I think my family and friends think everyone should get vaccinated against COVID-19. |
| Involvement | I think COVID-19 vaccines is an important topic. |
|  | I follow the news about COVID-19 vaccines. |
|  | I know much about COVID-19 vaccines. |
| Descriptive information** | How old are you? |
|  | What is your gender? |
|  | What is your level of education (your highest completed degree)? |
|  | Are you or have you been infected with the novel corona virus? |
|  | How is your health in general? |

*From wave 5 onwards, respondents were asked about their vaccination status, and the item on vaccination intention was skipped for respondents who reported to have received the vaccine. Also from wave 5 onwards. an item was added on an emerging public debate about granting different rights to vaccinated individuals (i.e., “People with a COVID-19 vaccination are more likely to regain their freedoms than people without vaccination”).

**The survey also contained items on residential area and migration background, but these variables were not integrated in the current manuscript.

**Instructions**

The information provided before the survey started covered the aim of the study (“*The aim of the study is to find out how people think about COVID-19 vaccines*”), the procedure (“*We will ask you several questions about your thoughts and feelings about the COVID-19 pandemic and COVID-19 vaccines. We also ask you questions about your health and behavior in this pandemic. There are no right or wrong answers: choose the answer that best reflects your opinion or feeling*.”), voluntary participation (“*If you now decide not to participate in this study, you will not be affected in any way. If you decide to stop during the research, you can do so at any time, without giving reasons and without this having any consequences for you. You can request to have your research data deleted within 7 days of the survey*.”), insurance (“*Standard liability insurance applies*”), privacy (“*Your privacy is guaranteed. Your personal data (about who you are) remains confidential and will not be shared without your permission. Your research data will be further analyzed by the researchers who collected the data. Research data published in scientific journals is anonymous and cannot be traced back to you. Fully anonymized research data can be made publicly available*.”), and contact information for questions or complaints. Finally, before completing the survey, we asked participants for consent to invite them for future measurements (“*We would like to receive your permission to invite you more often for this questionnaire. If you no longer wish to be invited, this will not affect the current questionnaire*”).

## Supplementary Note 9: R code and data

The R code version 4.1.2; ^3^ and data with codebook are provided on OSF (see .rmd file for script and Zip file for data): [https://osf.io/357h4/](https://osf.io/357h4/?view_only=a386a5e1288f435fabeba4aa8fbc5cb4)

*Versions packages*

rela_4.1

ggpubr_0.4.0

gridExtra_2.3

kableExtra_1.3.4

broom_0.7.10

NetworkComparisonTest_2.2.1

GPArotation_2014.11-1

psych_2.1.9

MPsychoR_0.10-8

caret_6.0-90

lattice_0.20-45

graphicalVAR_0.3

mlVAR_0.5

matrixcalc_1.0-5

openxlsx_4.2.4

Matrix_1.3-4

haven_2.4.3

janitor_2.1.0

mgm_1.2-12

bootnet_1.5

magrittr_2.0.1

igraph_1.2.8

qgraph_1.9

forcats_0.5.1

stringr_1.4.0

dplyr_1.0.7

purrr_0.3.4

readr_2.1.0

tidyr_1.1.4

tibble_3.1.6

ggplot2_3.3.5

tidyverse_1.3.1

psychonetrics_0.10

## Supplementary Note 10: Node construction with items per node

After collecting the data, items from the survey were translated into nodes for the broad COVID-19 vaccines network. Nodes were based on either a single item in the survey or composed from multiple items (i.e., mean score on items). Supplementary Table 10.1 shows each node with its construction approach. First, we excluded items that formed predetermined nodes. Next, we conducted Principal Axis Factoring (PAF) analyses to identify components in the data that formed separate nodes, and did so separately for items on attitudes toward COVID-19 vaccines, involvement in COVID-19 vaccines, and attitudes toward the COVID-19 pandemic. The PAF results are presented below. The selected rotation method was Oblimin due to the expected intercorrelation between items. Extraction of dimensions was based on eigenvalues greater than one. This analysis was conducted with the most inclusive dataset available, in which the datasets from the six waves were combined to include all respondents that finished the survey in one of the six waves (approximately 1500 respondents per wave with a total of 9051 respondents). Repeating this analysis with a dataset containing every wave from the longitudinal sample (744 respondents) and the dataset from only the first wave (1501 respondents) led to comparable node construction.^[[1]](#footnote-1)^ This implies that node construction is not affected by attrition during the course of the study. Detailed results from the node construction analyses are provided on OSF (see .html file): https://osf.io/357h4/

Supplementary Table 10.1 - Overview of nodes and the approach to the selection of items per node

| Nodes | Approach to combining items in node |
| --- | --- |
| General Attitude Vaccination | Predetermined single item node |
| Intention Vaccine | Predetermined single item node |
| Pandemic Negative Affect and Cognitions | PAF – Component identified in items on COVID-19 pandemic |
| Pandemic Trust Authorities | Predetermined single item node |
| Preventive Behaviors | PAF – Component identified in items on COVID-19 pandemic |
| Vaccines Attitude | PAF – Component identified in items on COVID-19 vaccines |
| Vaccines Involvement | PAF – Single component |
| Vaccines Negative Affect | PAF – Component identified in items on COVID-19 vaccines |
| Vaccines Social Norm | Predetermined – Two item construct |
| Vaccines Trust | Predetermined – Two item construct |

**Attitudes toward the COVID-19 pandemic**

This analysis included nine items on affect, cognition and behavior in regard to the COVID-19 pandemic (see Supplementary Table 10.2). Kaiser-Meyer-Olkin Measure of Sampling Adequacy was .86 and Bartlett’s test of sphericity was significant, χ²(36) = 32010.70, *p* < .001, supporting a rationale for performing PAF with these items. The results suggested two components: the first component contained items on negative affect and cognition regarding the COVID-19 pandemic, and the second component consisted of items on preventive behaviors during the COVID-19 pandemic. Although item 3 (‘*I worry about the impact the COVID-19 pandemic will have on society’*) showed a rather low component load, excluding the item did not improve scale reliability considerately (*a* = .83, if deleted *a* = .85), thus the item was maintained. The resulting nodes were *Pandemic Negative Affect and Cognitions*, consisting of six items (item 1 to 6 shown below; *a* = .83), and the node *Preventive Behaviors* that consisted of three items (item 7 to 9 shown below; *a* = .74).

Supplementary Table 10.2 – Pattern matrix of PAF with items on attitudes toward the COVID-19 pandemic

| No. | Survey items | Pandemic Negative Affect and Cognitions  Component load | Preventive  Behaviors Component load |
| --- | --- | --- | --- |
| 1 | I am afraid of getting infected with the corona virus. | .86 |  |
| 2 | I worry about losing friends or family to COVID-19. | .77 |  |
| 3 | I worry about the impact the COVID-19 pandemic will have on society. | .39 |  |
| 4 | COVID-19 is dangerous to my health. | .63 |  |
| 5 | COVID-19 is much more dangerous than the flu. | .45 |  |
| 6 | It is likely that I will get COVID-19. | .59 |  |
| 7 | I keep 1.5 meters away from others as much as possible. |  | .89 |
| 8 | I wash my hands often with soap and water. |  | .54 |
| 9 | I wear a face mask in public areas. |  | .67 |

**Attitudes toward COVID-19 vaccines**

This analysis included 16 items on affect, cognition and behavior in regard to COVID-19 vaccines (see Supplementary Table 10.3). Kaiser-Meyer-Olkin Measure of Sampling Adequacy was .96 and Bartlett’s test of sphericity was significant, χ²(120) = 120601.54, *p* < .001, supporting a rationale for performing PAF with these items. Results suggested two components: a component with positive affect and cognition items regarding COVID-19 vaccines, and a component consisting of negative affective items regarding COVID-19 vaccines. The low component load of item 4 (‘*I am afraid of the injection when vaccinated with COVID-19 vaccines*’) suggested to exclude this item, which was supported by an increase in scale reliability after deletion (four items a = .79, if item deleted a = .88). The item ‘*My religion or worldview has a lot of influence on my choice about vaccinating against COVID-19*’ was excluded because it loaded insufficiently on both components. This resulted in two nodes regarding COVID-19 vaccines: the node *Vaccines Negative Affect* consisting of 3 items (item 1 to 3 shown below; *a* = .88), and the node *Vaccines Attitude* consisting of 11 items (items 5 to 15 shown below; *a* = .95).

Supplementary Table 10.3 – Pattern matrix of PAF with items on attitudes toward COVID-19 vaccines. Items presented in italic were excluded from components.

| No. | Survey items | Vaccines Attitude Component load | Vaccines Negative Affect  Component load |
| --- | --- | --- | --- |
| 1 | I worry about the safety of COVID-19 vaccines. |  | .64 |
| 2 | I have mixed feelings about COVID-19 vaccines. |  | .56 |
| 3 | I feel misled about the safety of COVID-19 vaccines. |  | .59 |
| *4* | *I am afraid of the injection when vaccinated with COVID-19 vaccines.* |  | *.33* |
| 5 | I am hopeful about COVID-19 vaccines. | .80 |  |
| 6 | I have a good feeling about COVID-19 vaccines. | .77 |  |
| 7 | People who do not want to get vaccinated against COVID-19 make me angry. | .86 |  |
| 8 | COVID-19 vaccines protect well against COVID-19. | .77 |  |
| 9 | To stop the pandemic, it is important that most people get vaccinated against COVID-19. | .82 |  |
| 10 | COVID-19 vaccines are safe for one's health. | .77 |  |
| 11 | The side effects of COVID-19 vaccines have been sufficiently studied. | .65 |  |
| 12 | People without a COVID-19 vaccination should no longer be allowed everywhere. | .82 |  |
| 13 | By getting vaccinated against COVID-19, I am protecting others from COVID-19. | .79 |  |
| 14 | I encourage people to get vaccinated against COVID-19. | .82 |  |
| 15 | I avoid people who do not get vaccinated against COVID-19. | .81 |  |
| *16* | *My religion or worldview has a lot of influence on my choice about vaccinating against COVID-19.* |  |  |

**Involvement in COVID-19 vaccines**

This analysis included three items on involvement regarding COVID-19 vaccines (see Supplementary Table 10.4). Kaiser-Meyer-Olkin Measure of Sampling Adequacy was .63 and Bartlett’s test of sphericity was significant, χ²(3) = 7109.54, *p* < .001, supporting a rationale for performing PAF with these items. Results suggested one component with all items on involvement. Although item 1 (‘*I know much about COVID-19 vaccines’*) showed a rather low component load, excluding the item did not improve scale reliability considerately (*a* = .75, if deleted *a* = .77), thus the item was maintained. This resulted in the node *Involvement* with the three items shown below (*a* = .75).

Supplementary Table 10.4 – Factor matrix of PAF with items on involvement regarding COVID-19 vaccines

| No. | Survey items | Involvement  Component load |
| --- | --- | --- |
| 1 | I know much about COVID-19 vaccines. | .54 |
| 2 | I think COVID-19 vaccines is an important topic. | .67 |
| 3 | I follow the news about COVID-19 vaccines. | .93 |

## References

1 van Borkulo, C. D. *et al.* Comparing network structures on three aspects: A permutation test. *Psychological Methods* **Advance online publication**, doi:10.1037/met0000476 (2022).

2 van Borkulo, C. D. in *Symptom network models in depression research: From methodological exploration to clinical application* 249–257 (University of Groningen, Groningen, 2018).

3 R Core Team. *R: A language and environment for statistical computing*, <http://www.R-project.org/> (2013).

1. For the node *Vaccines Attitude*, repeating the analysis with the longitudinal sample resulted in two components when maintaining the extraction criteria of eigenvalues greater than one. Fixing the number of components according to the number of components observed in the other samples led to similar output for the *Vaccines Attitude* node. [↑](#footnote-ref-1)
